# Caregivers: the potential infection resources for the sustaining epidemic of hand, foot, and mouth disease/herpangina in Guangdong, China?

**DOI:** 10.1186/s13690-021-00574-8

**Published:** 2021-04-23

**Authors:** Jundi Liu, Yan Chen, Peipei Hu, Lin Gan, Qimin Tan, Xinqiao Huang, Zhanzhong Ma, Cuiji Lin, Dawei Wu, Xun Zhu, Dingmei Zhang

**Affiliations:** 1 School of Public Health, Sun Yat-sen University, Guangzhou, China; 2 Zhongshan Center for Diseases Prevention and Control, Zhongshan, China; 3 Medical College of Shaoguan University, Shaoguan, China; 4 Yonghe Community Health Service Center, Yongning Street, Zengcheng District, Guangzhou, China; 5 Clinical Laboratory, Yuebei People’s Hospital Affiliated to Shantou University Medical College, Shaoguan, China; 6 Department of Microbiology, Zhongshan School of Medicine, Sun Yat-sen University, Guangzhou, China

**Keywords:** Hand, foot, and mouth disease, Herpangina, Caregivers, Logistic regression analysis

## Abstract

**Background:**

Although several measures have been taken to control hand foot and mouth disease (HFMD) and herpangina (HA), these two diseases have been prevalent in China for 10 years with high incidence. We suspected that adults’ inapparent infection might be the cause of the continued prevalence of HFMD/HA infection in mainland China.

**Methods:**

To explore the role of adults (especially caregivers) in the transmission process of HFMD/HA among children, 330 HFMD/HA cases and 330 healthy children (controls) were selected for a case–control study. Then, data were analyzed by logistic regression.

**Results:**

Single-variable analyses revealed that caregivers who tested positive for enterovirus was a significant risk factor of HFMD/HA transmission to children (adjusted odds ratio (OR) = 9.22; 95% CI, 1.16 to 73.23). In the final multivariable model, caregiver behavior, such as cooling children’s food with mouth (OR = 1.85; 95% CI, 1.11 to 3.08) and feeding children with their own tableware (OR = 2.19; 95% CI, 1.07 to 4.45), significantly increased the risk of transmitting HFMD/HA to children. On the contrary, washing hands before feeding children reduced such risk.

**Conclusions:**

These results implied that the caregivers might be the infectious source or carriers of enterovirus. Therefore, preventing or treating the caregivers’ enterovirus infection and improving their hygiene habits, especially when they are in contact with children, could provide a breakthrough for the effective control of HFMD/HA.

## Background

Hand, foot, and mouth disease (HFMD) and herpangina (HA) are caused by human enterovirus, especially by serotypes of human enterovirus 71 (EV71), coxsackievirus A, and coxsackievirus B [1]. As reported, HFMD/HA epidemics predominantly occur in the Asia–Pacific region [2], such as South Korea [3], Thailand [4], Vietnam [5]. For most cases, the symptoms of HFMD/HA are mild and self-limiting. However, severe complications, including myocarditis, acute flaccid paralysis, and even death, occur in a few cases [6, 7]. In China, HFMD has been a notifiable disease in 2008. From January 2004 to December 2013, the incidence of HFMD has ranked third in the incidence of infectious diseases [8]. Although the EV71 vaccine has been successfully licensed in 2016, approximately 2 million cases of HFMD are reported in China every year [9]. Thus, the prevalence of HFMD/HA in China remains severe.

At present, most studies on HFMD/HA suggest that nurseries and kindergartens are the primary sites for the transmission of infection [10]. Therefore, the prevention and control of HFMD/HA is mainly to take secondary prevention measures, that is, early detection, early report, early diagnosis, early treatment, and early isolation. During an HFMD/HA epidemic, children in nurseries and kindergartens should be screened in the morning, and suspected cases should be reported, isolated, and treated in time. In case of an HFMD/HA outbreak, measures are taken to close classes or kindergartens in strict accordance with school suspension standards. A series of measures plays a good regulating role in preventing the further spread of HFMD/HA among children living collectively [11, 12], thus greatly reducing the number of cases of mutual infection. However, among the 2 million reported cases of HFMD, children aged 1–2 have the highest risk of disease although they are generally scattered children who have no experience of living in nurseries or kindergartens. In addition, most of them have no clear history of patient contact. Therefore, how to reduce these primary infections has become the key issue to control the HFMD/HA epidemic in China.

Generally, the HFMD/HA epidemic can only be controlled effectively by containing HFMD/HA from the source, i.e., identifying the exact source and route of transmission. Consequently, different methods have been used in related studies. For example, Lu et al. [13] showed that children’s hospitals may have a crucial role in HFMD transmission. Wang et al. [14] established a mathematical model to illustrate the important role of asymptomatic infection and contaminated environments in new HFMD infections. In addition, the caregivers may have had contact with children with HFMD/HA or article surfaces with enterovirus attachment; then, as a vector of the enterovirus, transmit the virus to their children. Studies have also found that hand washing by caregivers is a protective factor for HFMD among children [15, 16], suggesting that transmission from adults to children is possible.

Hence, a case–control study was conducted to explore the role of caregivers in the transmission mechanism of HFMD/HA by analyzing data obtained from the cities of Guangzhou and Shaoguan. Thus, a theoretical basis for the prevention and control strategies and measures of HFMD/HA is provided from the perspective of caregivers.

## Method

### Study population

This study was conducted in the community health service center in Yonghe, Yongning Street, Zengcheng District, Guangzhou City and the Yuebei People’s Hospital of Shaoguan City between May 1, 2019 and June 30, 2019. All children aged five years old or younger and their caregivers who volunteered for the study were chosen by using the convenient sampling method. Then, throat swab samples of the caregivers were collected and stored at − 80 °C. In addition, the caregivers were asked to complete a study questionnaire.

### Case and control groups

The clinical diagnosis criteria of HFMD cases included children who have the symptoms with fever; scattered herpes in oral mucosa; maculopapular rash and herpes in hands, feet, and buttocks; and inflammatory erythema around herpes. Moreover, the diagnosis criteria of herpangina cases included children who have the signs with anorexia, high fever, salivation, and vomiting; moreover, pale herpes could be observed in the soft palate, pharyngeal palatal arch, and the mucosa of the uvula. Children who had the above signs and were diagnosed with HFMD/HA by clinicians were defined as cases in the study. Furthermore, the cases were collected from the pediatric clinic of the Yonghe community health service center and the Yuebei People’s Hospital. A total of 330 cases were selected.

The control group in the study included healthy children who were recruited from the vaccination clinics of the Yonghe community health service center. Moreover, the control group had not suffered from HFMD/HA in the past months. All the children’s ages were divided into < 6 months old, ~ 6 months old, ~ 12 months old, ~ 24 months old, ~ 36 months old, ~ 48 months old, 60–71 months old. Then, 330 control–children who were matched to the case–children in terms of frequency by age group were selected.

### Questionnaire

The questionnaire used in the study covered demographic information of the children (e.g., children’s age) and the caregivers (e.g., caregivers’ age; hygiene habits, such as washing hands before meals; behavior when looking after children, such as washing hands before feeding children). In order to ensure the validity of the questionnaire, the first draft of the questionnaire was designed through literature review, and then the final draft was determined through expert discussion and pre-investigation. All interviewers were systematically trained.

### Laboratory methods

The throat swab specimens of caregivers were defrosted in a − 4 °C environment. Each 200 μl specimen was processed by using LogPure Viral RNA/DNA Kits (250 reactions) (Magen, Guangzhou, China). Lastly, 60 μl of viral RNA was extracted. Reverse transcription was conducted using a HiScript® II Q Select RT SuperMix for qPCR kit (Vazyme, Nanjing, China). The primers for enterovirus were designed on the basis of other literature [17, 18]. Table 1 lists the details of the primers for enterovirus (Invitrogen Custom Primers, Shanghai). Next, 10 μl of 2 × qPCR mix (iQ™ SYBR® Green Supermix, Bio-Rad, America), 1 μl of forward primer, 1 μl of reverse primer, 1 μl of probe, 3 μl of tri-distilled water and 4 μl s of cDNA sample were added into each PCR tube. Afterwrad, Taqman real-time PCR was conducted with a quantitative fluorescence analyzer (Bio-rad CFX96, America). All experiments were performed in accordance with the manufacturer’s instruction. When the Ct value was greater than 20 or less than 40, corresponding specimens were considered positive.

**Table 1:** Primers for the enterovirus real-time fluorescence quantitative PCR

Primer Name	Sequence 5′-3’	3′ Label	5′ Label	Size(bp)
**Forward primer**	CCCTGAATGCGGCTAATCC			
**Reverse primer**	ATTGTCACCATAAGCAGCCA			
**Probe**	AACCGACTACTTTGGGTGTCCGTGTTTC	BHQ1	FAM	146

### Statistical analysis

Mean (SD) was used for continuous data, and categorical data were expressed as frequency (percentage, %). Logistic regression analysis was used to analyze the potential risk factors of HFMD/HA infection among children by using SPSS ver. 25. Initially, statistically significant predictors were screened by single variable analysis. Then, the statistically significant variables were place into the multivariable model during stepwise regression. Moreover, children’s age as the control variable was placed into the multivariable logistic regression model. Odds ratios (ORs) and 95% confidence intervals (95% CIs) were calculated to determine whether a variable is a risk factor of HFMD/HA infection in children. *P* < 0.1 indicated statistical significance in single-variable analysis, whereas *P* < 0.05 indicated statistical significance in the multivariable model. Stratified analysis was further performed to assess whether the associations between the variables and HFMD/HA among children were modified by demographic characteristics, such as children’s age. Moreover, 0.05 was chosen as the level of significance (*P* < 0.05).

## Results

As shown in Tables 2, 27.6% (91/330) of the cases were aged between 12 and 23 months old, and the cases aged under 6 months old had the lowest rate with 2.1% (7/330); the same goes for the control group. Moreover, compared with children whose family monthly income was less than RMB 5000, children whose family monthly income was RMB 10000–30,000 and RMB 30000–50,000 had a higher risk of contracting HFMD/HA. In addition, compared with children whose families had no other children under five, children whose families had other children aged five and under were more likely to contract HFMD/HA (OR = 1.38; 95% CI, 1.01 to 1.87). However, having extra-curricular classes was a protective factor (OR = 0.54; 95% CI, 0.33 to 0.89) for HFMD/HA suffering in children. Otherwise, children’s gender, children’s current residence, and scattered children or not did not significantly differ between the case and control groups.

**Table 2:** Demographic characteristic of the children in cases and controls

Characteristic	Cases(n/%) (***n*** = 330)	Controls(n/%) (n = 330)	OR (95%CI)	***P***-Value
**Children’s age (month old)**				–
< 6	7 (2.1)	7 (2.1)	–	
6–11	36 (10.9)	36 (10.9)	–	
12–23	91 (27.6)	91 (27.6)	–	
24–35	58 (17.6)	58 (17.6)	–	
36–47	53 (16.1)	53 (16.1)	–	
48–59	55 (16.7)	55 (16.7)	–	
60–71	30 (9.1)	30 (9.1)	–	
**Children’s gender**				0.754
Male	182 (55.2)	186 (56.4)	1(reference)	
Female	148 (44.8)	144 (43.6)	1.05 (0.77–1.43)	
**Children’s current residence**				0.111
Rural	119 (36.1)	139 (42.1)	1(reference)	
Urban	211 (63.9)	191 (57.9)	1.29 (0.94–1.77)	
**Children’s family monthly income (¥)**				< 0.001^*^
< 5000	29 (8.8)	69 (21.0)	1(reference)	
5000~	55 (16.7)	129 (39.2)	1.01 (0.59–1.74)	
10,000~	193 (58.5)	90 (27.4)	5.10 (3.09–8.42)	
30,000~	45 (13.6)	15 (4.6)	7.14 (3.45–14.78)	
≥ 50,000	8 (2.4)	26 (7.9)	0.73 (0.30–1.81)	
**Scattered children**				0.273
Yes	189 (57.3)	175 (53.0)	1(reference)	
No	141 (42.7)	155 (47.0)	0.84 (0.62–1.15)	
**Other children aged five and under in children’s family**				0.042^*^
No	168 (50.9)	194 (58.8)	1(reference)	
Yes	162 (49.1)	136 (41.2)	1.38 (1.01–1.87)	
**Having extra-curricular class**				0.015^*^
No	303 (91.8)	283 (85.8)	1(reference)	
Yes	27 (8.2)	47 (14.2)	0.54 (0.33–0.89)	

^*^Significance difference between two groups where the *P*-value is less than 0.1

As shown in Table 3, caregivers’ age was positively associated with HFMD/HA among children (*P* < 0.1). Meanwhile, caregivers with a high education level could reduce the possibility of HFMD/HA among children. Compared with that of caregivers with primary education or below, the OR for HFMD/HA among children whose caregivers had junior high school education, senior high school education, college education, university education, and master education or above was 0.49, 0.45, 0.22, 0.37, and 0.50, respectively. In addition, the relationship between the children and caregivers was also associated with HFMD/HA among children. Compared with caregivers who were fathers, caregivers who were grandfathers or grandmothers significantly increased the risk of HFMD/HA among children (OR = 2.33; 95% CI, 1.31 to 4.13). Nevertheless, no statistical difference was found between fathers and mothers (OR = 0.86; 95% CI, 0.55 to 1.32). In addition, caregivers’ gender was not significantly different between the two groups.

**Table 3:** Demographic characteristics of the caregivers in the case and control groups

Characteristic	Cases(n/%) (***n*** = 330)	Controls(n/%) (***n*** = 330)	OR (95%CI)	***P***-Value
**Caregivers’ age(mean ± SD)**	37.82 ± 11.46	33.74 ± 8.48	1.04 (1.03–1.06)	< 0.001^*^
Caregivers’ gender				0.921
Male	63 (19.1)	62 (18.8)	1(reference)	
Female	267 (80.9)	268 (81.2)	0.98 (0.66–1.45)	
**Caregivers’ education**				0.001^*^
Primary school or below	36 (10.9)	15 (4.5)	1(reference)	
Junior high school	99 (30.0)	84 (25.5)	0.49 (0.25–0.96)	
Senior high school	90 (27.3)	83 (25.2)	0.45 (0.23–0.89)	
College degree	43 (13.0)	80 (24.2)	0.22 (0.11–0.45)	
University degree	56 (17.0)	63 (19.1)	0.37 (0.18–0.75)	
Master degree or above	6 (1.8)	5 (1.5)	0.50 (0.13–1.89)	
**The caregiver’s relationship with the children**				< 0.001^*^
Father	49 (14.8)	50 (15.2)	1(reference)	
Mother	208 (63.0)	248 (75.2)	0.86 (0.55–1.32)	
Grandpa or grandma	73 (22.1)	32 (9.7)	2.33 (1.31–4.13)	

^*^Significance difference between two groups where the *P*-value is less than 0.1

In the single-variable analyses of caregivers’ hygiene habits (Table 4), “washing of hands of caregivers after using the bathroom” was not associated with HFMD/HA among children (*P* = 0.507). Compared with that of “almost always washing hands before meals,” the OR for “sometimes washing hands before meals” was 0.74 (95% CI, 0.55 to 1.02), and that for “never washing hands before meals” was 9.78 (95% CI, 1.25 to 76.62). In addition, “washing hands after going out” was a protective factor of HFMD/HA among children (OR = 0.66; 95% CI, 0.48 to 0.91). Moreover, a similar HFMD/HA prevention effect could be found in “using soaps or hand sanitizers,” “washing hands for 20 seconds,” and “wiping hands after washing hands;” the ORs were 0.43 (95% CI, 0.32 to 0.59), 0.32 (95% CI, 0.23 to 0,45) and 0.50 (95% CI, 0.34 to 0.75), respectively.

**Table 4:** Single variable analyses of association of caregivers’ hygiene habits with children’s HFMD/HA suffering

Predictor	Cases(n/%) (***n*** = 330)	Controls(n/%) (***n*** = 330)	Unadjusted OR (95%CI)	***P***-Value
**Washing hands before meals**				0.012^*^
Almost always	171 (51.8)	152 (46.2)	1(reference)	
Sometimes	148 (44.8)	176 (53.5)	0.74 (0.55–1.02)	
Never	11 (3.3)	1 (0.3)	9.78 (1.25–76.62)	
**Washing hands after defecation**				0.507
Sometimes	9 (2.7)	12 (3.6)	1.35 (0.56–3.24)	
Almost always	321 (97.3)	318 (96.4)	1(reference)	
**Washing hands after going out**				0.012^*^
No	133 (40.3)	102 (30.9)	1(reference)	
Yes	197 (59.7)	228 (69.1)	0.66 (0.48–0.91)	
**Using soap or hand sanitizer**				< 0.001^*^
No	220 (66.7)	153 (46.4)	1(reference)	
Yes	110 (33.3)	177 (53.6)	0.43 (0.32–0.59)	
**Washing hands for 20 s**				< 0.001^*^
No	247 (74.8)	161 (48.8)	1(reference)	
Yes	83 (25.2)	169 (51.2)	0.32 (0.23–0.45)	
**Wiping hands after washing hands**				0.001^*^
No	82 (24.8)	47 (14.2)	1(reference)	
Yes	248 (75.2)	283 (85.8)	0.50 (0.34–0.75)	

^*^Significance difference between two groups where the *P*-value is less than 0.1

As shown in Table 5, children whose caregivers tested positive for enterovirus were more likely to contract the disease (OR = 9.22; 95% CI, 1.16 to 73.23). Meanwhile, compared with “never kissing the children per day,” “kissing the children 1–3 times per day” (OR = 1.59; 95% CI, 1.13 to 2.24), “kissing the children 4–6 times per day” (OR = 2.57; 95% CI, 1.34 to 4.94) and “kissing the children more than 6 times per day” (OR = 2.37; 95% CI, 1.26 to 4.47) were risk factors of HFMD/HA among children. In addition, “washing hands before feeding the children,” “washing hands after defecating,” “preparing special utensils and towels for children,” “cooling children’s food with their mouth,” “feeding the children with their own tableware,” “disinfecting the tableware and bottles,” “disinfecting the toys,” “the number of times a week children are taken out,” and “the average time of outdoor activities for children every day” contributed to children’s HFMD/HA infection. However, no significant association was found between “caregivers and children sleeping in the same bedroom” and HFMD/HA among children.

**Table 5:** Single variable analyses of the association between caregivers’ behaviors with children’s HFMD/HA suffering

Predictor	Cases(n/%) (n = 330)	Controls(n/%) (n = 330)	Unadjusted OR (95%CI)	***P***-Value
**Caregiver’s enterovirus**				0.048^*^
Negative	322 (97.6)	329 (99.7)	1(reference)	
Positive	9 (2.7)	1 (0.3)	9.22 (1.16–73.23)	
**Number of kissing the children per day**				0.003^*^
0	88 (26.7)	128 (38.8)	1(reference)	
1–3	181 (54.8)	166 (50.3)	1.59 (1.13–2.24)	
4–6	30 (9.1)	17 (5.2)	2.57 (1.34–4.94)	
> 6	31 (9.4)	19 (5.8)	2.37 (1.26–4.47)	
**Sleeping in the same bedroom as the children**				0.798
No	33 (10.0)	35 (10.6)	0.94 (0.57–1.55)	
Yes	297 (90.0)	295 (89.4)	1(reference)	
**Washing hands before feeding children**				< 0.001^*^
Almost always	140 (42.4)	166 (50.3)	0.07 (0.03–0.19)	
Sometimes	133 (40.3)	159 (48.2)	0.07 (0.03–0.19)	
Never	57 (17.3)	5 (1.5)	1(reference)	
**Washing hands after defecating the children**				< 0.001^*^
Almost always	165 (50.0)	176 (53.3)	0.10 (0.05–0.22)	
Sometimes	107 (32.4)	146 (44.3)	0.13 (0.06–0.28)	
Never	58 (17.6)	8 (2.4)	1(reference)	
**Special utensils and towels for children**				0.002^*^
No	62 (18.8)	34 (10.3)	1(reference)	
Yes	268 (81.2)	296 (89.7)	0.50 (0.32–0.78)	
**The way to cool food**				< 0.001^*^
Natural cooling	108 (32.7)	216 (65.5)	1(reference)	
Cooling with mouth	222 (67.3)	114 (34.5)	3.90 (2.82–5.38)	
**Feeding the children with the caregiver’s own tableware**				< 0.001^*^
Almost always	142 (43.0)	36 (10.9)	4.45 (2.83–6.99)	
Sometimes	86 (26.1)	179 (54.2)	0.54 (0.37–0.79)	
Never	102 (30.9)	115 (34.8)	1(reference)	
**Disinfecting tableware and bottles**				0.003^*^
No	62 (18.8)	35 (10.6)	1(reference)	
Yes	268 (81.2)	295 (89.4)	0.51 (0.33–0.80)	
**Disinfecting toys**				< 0.001^*^
No	259 (78.5)	88 (26.7)	1(reference)	
Yes	71 (21.5)	242 (73.3)	0.10 (0.07–0.14)	
**Number of times a week to take children out**				< 0.001^*^
0	34 (10.3)	91 (27.6)	1(reference)	
1	62 (18.8)	156 (47.3)	1.06 (0.65–1.74)	
2	36 (10.9)	36 (10.9)	2.68 (1.46–4.91)	
3	16 (4.8)	16 (4.8)	2.68 (1.21–5.94)	
≥ 4	182 (55.2)	31 (9.4)	15.71 (9.09–27.18)	
**Average time of outdoor activities for children every day (hour)**				0.001^*^
< 1	54 (16.4)	66 (20.0)	1(reference)	
1	153 (46.4)	172 (52.1)	1.08 (0.71–1.66)	
2–3	85 (25.8)	82 (24.8)	1.27 (0.79–2.03)	
≥ 4	38 (11.5)	10 (3.0)	4.64 (2.12–10.17)	

^*^Significance difference between two groups where the *P*-value is less than 0.1

Children’s ages, which were matching variables, and the variables that were statistically significant through single-variable analyses were included in the multivariable model (Table 6). Then, the final multivariable model identified the monthly income of RMB 10000–30,000 (OR = 5.43; 95% CI, 2.61 to 11.32) and RMB 30000 ~ 50,000 (OR = 16.38; 95% CI, 5.59 to 47.99), “cooling children’s food by blowing with their mouth” (OR = 1.85; 95% CI, 1.11 to 3.08), “always feeding the children with their own tableware” (OR = 2.19; 95% CI, 1.07 to 4.45), “taking children out twice a week” (OR = 3.87; 95% CI, 1.67 to 8.99), “taking children out thrice a week” (OR = 3.85; 95% CI, 1.34 to 11.08), “taking children out four times a week or more” (OR = 14.34; 95% CI, 6.97 to 29.49), and “an average of 4 hours and more of outdoor activities for children every day” (OR = 5.74; 95% CI, 1.78 to 18.49) as independent risk factors of HFMD/HA among children. By contrast, “having extra-curricular classes” (OR = 0.40; 95% CI, 0.17 to 0.95), “caregivers with college education level or above,” “washing hands before feeding the children” and “disinfecting the toys” (OR = 0.12; 95% CI, 0.07 to 0.21) were protective factors for HFMD/HA among children.

**Table 6:** Multi-variable analyses of predictors for HFMD/HA suffering in children

Predictor	Adjusted OR	95% CI	***P***-Value
**Children’s family month income (¥)**			< 0.001^*^
< 5000	1	reference	
5000~	0.77	0.37–1.62	0.492
10,000~	5.43	2.61–11.32	< 0.001^*^
30,000~	16.38	5.59–47.99	< 0.001^*^
≥ 50,000	1.39	0.34–5.63	0.643
**Having extra-curricular class**			
No	1	reference	
Yes	0.40	0.17–0.95	0.039^*^
**Caregivers’ education**			0.003^*^
Primary school and below	1	reference	
Junior high school	0.55	0.19–1.59	0.270
Senior high school	0.71	0.25–1.98	0.512
College degree	0.25	0.09–0.74	0.012^*^
University degree	0.21	0.07–0.64	0.006^*^
Master degree or above	0.10	0.01–0.76	0.026^*^
**Washing hands before feeding children**			0.010^*^
Almost always	0.20	0.06–0.70	0.011^*^
Sometimes	0.15	0.05–0.52	0.003^*^
Never	1	reference	
**The way to cool food**			
Natural cooling	1	reference	
Cooling by blowing with mouth	1.85	1.11–3.08	0.018^*^
**Feeding the children with the caregiver’s own tableware**			0.006^*^
Almost always	2.19	1.07–4.45	0.031^*^
Sometimes	0.74	0.42–1.29	0.288
Never	1	reference	
**Disinfecting toys**			
No	1	reference	
Yes	0.12	0.07–0.21	< 0.001^*^
**Number of times a week to take children out**			< 0.001^*^
0	1	reference	
1	1.71	0.89–3.29	0.266
2	3.87	1.67–8.99	0.005^*^
3	3.85	1.34–11.08	0.027^*^
≥ 4	14.34	6.97–29.49	< 0.001^*^
**Average time of outdoor activities for children every day (hour)**			0.030^*^
< 1	1	reference	
1	1.24	0.65–2.37	0.523
2–3	1.43	0.68–3.01	0.349
≥ 4	5.74	1.78–18.49	0.003^*^

^*^Significance difference between two groups where the *P*-value is less than 0.05

The results of stratified analysis by children’s age (under 3 years old and 3 years old and above) could be seen in Table 7. Regardless of children’s age, no statistical associations were found between having extracurricular class and HFMD/HA among children in the single-variable analysis model (*P* > 0.05). The same results could be found in the multivariable analysis model.

**Table 7:** Associations between having extra-curricular class and children’s HFMD/HA suffering by children’s age

Children’s age	Having extra-curricular class	Cases (n/%)	Controls (n/%)	OR^**a**^ (95%CI)	OR^**b**^ (95%CI)	***P***^**a**^	***P***^**b**^
< 3	No	192 (100)	181 (94.27)	1(reference)	1(reference)	0.999	0.999
	Yes	0 (0)	11 (5.73)	< 0.001(−)	< 0.001(−)		
≥3	No	111 (80.43)	102 (73.91)	1(reference)	1(reference)	0.198	0.265
	Yes	27 (19.57)	36 (26.09)	0.69 (0.39–1.22)	0.62 (0.33–1.18)		

^a^Single variable analysis

^b^Adjusted for children’s age, children’s current residence, children’s family monthly income, other children aged five and under in children’s family

## Discussion

HFMD/HA is generally regarded as children’s disease; thus, studies have focused on children aged five and under. Although adults could contract HFMD/HA, researchers have not paid enough attention to this group [19, 20]. Nevertheless, the findings of the present study suggested that adults, especially caregivers, might be the latent infectious source or carriers of children’s HFMD/HA.

In this study, nine (2.7%) caregivers in the case group and one (0.3%) caregiver in the control group tested positive for enterovirus. The single-variable analysis also showed that the caregiver who tested positive for enterovirus was a significant risk factor of HFMD/HA among children. However, after controlling for the age group and other factors, this factor (caregivers with enterovirus infection) was not included in the multivariable model because the number of caregivers who tested positive for enterovirus was too small. In our previous study, the positive rate for enterovirus of children’s caregivers was 7.14% [17]. Another previous study reported that the positive rate for enterovirus of sick children’s close contacts was 14.1% [21]. Furthermore, in consideration of the nondifferential misclassification bias between the case and control groups, the conclusion that caregivers who test positive for enterovirus increase the possibility of HFMD/HA among children is unchanged. Moreover, the inference that the caregivers probably transmit enterovirus to their children and even to other children they contacted with was reasonable [22, 23].

The study showed that compared with a monthly family income of less than RMB 5000, a monthly family income of RMB 10000–30,000 and RMB 30000–50,000 increased the risk of HFMD/HA infection in children. This result contradicts Chen’s study [24] probably because the present study was conducted in hospitals rather than in a village clinic, where high-income families preferred to see doctors for their children.

Taking “caregivers with primary education or below” as reference, “caregivers with college education or above” could reduce children’s risk of HFMD/HA; Chen’s study revealed a similar finding [24, 25]. Similar to other studies, the present study found that the higher the level of caregivers’ education, the richer the knowledge of HFMD/HA and the higher the awareness of acquiring new knowledge through various channels [26]. Meanwhile, our study revealed that having extra-curricular classes was a protective factor of HFMD/HA in children on the basis of the multi-variable non-conditioned logistic regression model. Generally, children who had extra-curricular class were school-aged children, not children under three years old who were susceptible to HFMD/HA [27, 28]. However, the difference of having extra-curricular classes between two groups had no significant when it was analysed with stratification according to children’s age. Therefore having extra-curricular class was not a significant influence factor of HFMD/HA among children.

This study also reveals that factors, such as “cooling children’s food by blowing with their mouth” and “always feeding the children with their own tableware,” increase risk of HFMD/HA among children. By contrast, “washing hands before feeding the children”and “disinfecting the toys” are protective factors. These findings are consistent with those of other studies [29–31]. The reason could be that although majority of adults infected with enterovirus are mostly recessive and healthy carriers, they can be the source of infection. In addition, enterovirus could also be transmitted through contact with respiratory secretions or feces of infected persons or contaminated hands or objects. All the results showed that the caregivers may be the infectious source or carriers of enterovirus in children. At the same time, children’s living habits and behavior are easily affected by their families and environment. Moreover, the health habits of children mainly depend on their caregivers. Therefore, to control HFMD/HA, caregivers’ enterovirus infection must be prevented, and some poor health habits, such as cooling children’s food by blowing with their mouth, must be stopped.

Lastly, this study also found that taking children out twice a week or more and an average of 4 h and more of outdoor activities for children every day increase the likelihood of contracting HFMD/HA among children. The studies of Feng et al. [15] and Xiang et al. [32] support our findings. A possible reason is that in crowded public places, pathogens could be carried by different individuals, making children susceptible to enterovirus infection. Therefore, caregivers should avoid taking children to crowded places or shorten the time outside with their children during an HFMD/HA epidemic.

This study has some limitations. First, most of the questionnaire content was about the survey of the healthy habits of the caregivers and thus was mainly based on the subjective judgment of the caregivers. Moreover, the caregivers intentionally exaggerated or minimized information about their health habits, leading to information bias. To control the information bias, all interviewers were professionally trained. Second, our study suggested that caregivers who tested positive for enterovirus were associated with HFMD/HA among children; however, a causal link between the two could not be established through a case–control study for the infection sequence of children and their caregivers could not be distinguished. In the future, a related cohort study should be conducted.

## Conclusion

This study suggested that children’s caregivers with latent enterovirus infection might become a potential source of HFMD/HA among children. To control the sustaining epidemic of HFMD/HA in Guangdong, China, some approaches should be taken to prevent the transmission of caregivers’ enterovirus infection to children.

## Data Availability

All data generated or analyzed during this study are included in the article. The datasets used and/or analysed during the current study are available from the corresponding author on reasonable request.

## Abbreviations

HFMD: Hand foot and mouth disease
HA: Herpangina
EV71: Human enterovirus 71
cDNA: Complementary Deoxyribonucleic Acid
DNA: Deoxyribonucleic Acid
RNA: Ribonucleic Acid
Ct: Cycle threshold
μl: Microliter
PCR: Polymerase Chain Reaction
OR: Odds ratio
